# Influence of organizational role, consensus and innovation status on perceived facilitators and barriers to adoption of innovative and evidence-based practices in state-supported mental health clinics

**DOI:** 10.1186/1748-5908-10-S1-A41

**Published:** 2015-08-20

**Authors:** Lawrence A Palinkas, Serene Olin, Brian Chor, Mee Young Um, Chung Hyeon Jeong, Briannon O'Connor, Sarah M Horwitz, Kimberly Hoagwood

**Affiliations:** 1 Department of Children, Youth and Families, School of Social Work, University of Southern California, Los Angeles, CA 90089-0411, USA; 2 Department of Child and Adolescent Psychiatry, New York University School of Medicine, New York, NY 10016, USA

## Introduction

Although D&I models identify several potential facilitators and barriers to adoption of evidence-based practices, the contextual characteristics that determine the relative importance of these factors remain largely unknown. The objectives of this study were to identify the most significant facilitators and barriers to innovation and adoption of EBPs as determined by agency and program directors; explore whether facilitators and barriers identified are associated with agency role and pace of adoption; and explore whether consensus on barriers is associated with pace of adoption.

## Methods

Semi-structured interviews were conducted with agency and program directors of 36 New York State-supported mental health clinics. Agencies were classified based on level of participation in different implementation activities ranging from no engagement to participation in a webinar, in-person training, or learning collaboratives. Data were analyzed using grounded theory analytic methods. Intra-organizational consensus was defined as the number of barriers identified by both types of directors divided by the total number of unique barriers.

## Findings

Three interconnected themes relating to barriers and facilitators were identified: costs associated with adoption, capacity for adoption, and acceptability of new practices. Agency directors were more concerned about financial and organizational capacity, and client buy-in and fit, while program directors were more concerned about time for training and staff buy-in. Concerns about cost, time for training, client buy-in, and organizational capacity were inversely associated and concern about reimbursement was positively associated with pace of adoption. Agency consensus on barriers and facilitators was associated with pace of innovation.

## Implications for D&I

Organizations assess EBPs and other innovations in terms of costs, capacity and acceptability. This assessment varies by role within an organization and by an organization's level of engagement in implementation activities. Consensus within an organization as to specific facilitators and barriers is a potential predictor of EBP adoption and implementation.

## Funding

NIH-NIMH P30 MH090322.

